# Molecular characterization of metastatic penile squamous cell carcinoma in developing countries and its impact on clinical outcomes: LACOG 2018 translational study

**DOI:** 10.1093/oncolo/oyae220

**Published:** 2024-09-02

**Authors:** Fernando Sabino Marques Monteiro, Antonio Machado Alencar Junior, Karine Martins da Trindade, Taiane Francieli Rebelatto, Fernando C Maluf, Antonia A Gazzola, Pablo M Barrios, Joaquim Bellmunt, Rafaela Gomes de Jesus, Gyl Eanes Barros Silva, Antonio Augusto Lima Teixeira Junior, Philippe E Spiess, Andre P Fay

**Affiliations:** Latin American Cooperative Oncology Group (LACOG), Porto Alegre, Brazil; Hospital Sírio Libanês, Oncology and Hematology Department, Brasilia, Brazil; Pontifícia Universidade Católica do Rio Grande do Sul (PUCRS), School of Medicine, Porto Alegre, Brazil; Latin American Cooperative Oncology Group (LACOG), Porto Alegre, Brazil; Hospital Universitário da Universidade Federal do Maranhão, Oncology Department, São Luis, Brazil; Latin American Cooperative Oncology Group (LACOG), Porto Alegre, Brazil; Instituto de Ensino e Pesquisa do Ceará, Fortaleza, Brazil; Latin American Cooperative Oncology Group (LACOG), Porto Alegre, Brazil; Latin American Cooperative Oncology Group (LACOG), Porto Alegre, Brazil; Hospital Israelita Albert Einstein, Oncology and Hematology Department, São Paulo, Brazil; Pontifícia Universidade Católica do Rio Grande do Sul (PUCRS), School of Medicine, Porto Alegre, Brazil; Latin American Cooperative Oncology Group (LACOG), Porto Alegre, Brazil; Dana Farber Cancer Institute and IMIM Research Lab, Harvard Medical School, Boston, United States; Latin American Cooperative Oncology Group (LACOG), Porto Alegre, Brazil; Hospital Universitário da Universidade Federal do Maranhão, Oncology Department, São Luis, Brazil; Postgraduate Program in Genetics, Ribeirão Preto Medical School, Universisty of São Paulo, Ribeirão Preto, Brazil; Department of GU Oncology, Moffitt Cancer Center, Tampa, United States; Latin American Cooperative Oncology Group (LACOG), Porto Alegre, Brazil; Pontifícia Universidade Católica do Rio Grande do Sul (PUCRS), School of Medicine, Porto Alegre, Brazil; Hospital Nora Teixeira, Oncology and Hematology Department, Porto Alegre, Brazil

**Keywords:** penile carcinoma, metastatic disease, genomic profile, molecular alterations

## Abstract

**Background:**

Penile squamous cell carcinoma (PSCC) is a rare malignancy. However, in developing countries the incidence rate is higher. The understanding of molecular alterations is essential for evaluating possible targets for more effective systemic therapies.

**Methods:**

We retrospectively collected clinical data of metastatic PSCC (mPSCC) patients who had received at least one prior systemic treatment from 3 Brazilian hospitals. Tumor samples were evaluated using the next-generation sequencing (NGS) Foundation One DX and immunohistochemistry (IHC). The objective was to identify and describe somatic genomic alterations known to be functional or pathogenic and their association with survival outcomes.

**Results:**

Twenty-three patients were identified, 22 and 18 patients had tumor samples analyzed by IHC and NGS, respectively. PD-L1 expression (CPS ≥ 1%) was positive in 14 patients (63.6%). Regarding the genomic alterations, 16 patients (88.9%) had some clinically relevant genomic alterations. *TP53, TERT, CDKN2A, PIK3CA*, *NOTCH1,* and CDKN2B loss were identified in 66.7%, 50%, 50%, 33.3%, 27.8%, and 22.2% of the patients, respectively. No MSI or TMB high (≥10 mutations/MB) cases were identified. *NOTCH1* mutation was identified only in HPV-negative patients and it was associated with worse OS (yes: 5.5 vs no: 12.8 months, *P* = .049) and progression-free survival (yes: 5.5 vs no: 11.7 months, *P* = .032).

**Conclusion:**

This study demonstrated that molecular alterations in mPSCC from developing countries are similar to those from developed countries. Predictive biomarkers for immunotherapy response such as TMB high or MSI were not identified. Specific gene mutations may identify patients with worse prognoses and open new avenues for therapeutic development.

## Teaser

Understanding molecular alterations is essential for evaluating possible targets for more effective systemic therapies for the rare malignancy penile squamous cell carcinoma.

**Implications for practice**

The identified genomic and molecular profile is an opportunity for clinical trials evaluating new treatment options for this rare malignant neoplasm that has been so far deprived of effective systemic treatments. In this context, this study identified potentially actionable molecular alterations that should be evaluated in further trials looking for new treatment options in this neoplasm. Furthermore, the genomic profile demonstrated in this study raises concerns regarding the effectiveness of immunotherapy.

## Introduction

Penile squamous cell carcinoma (PSCC) is a rare disease with an age-standardized incidence rate (ASR) of 0.8 case per 100 000 men corresponding to 0.2% of cancer cases globally.^1^ However, in developing regions such as Africa, Eastern Europe, and Latin and Central America the incidence of PSCC is higher over the rest of the world.^2^ In this context, Brazil has one of the highest incidences of PSCC in the world with an ASR of six cases per 100 000 men.^3^ This high incidence rate is probably due to the low income and educational level of these regions in addition to the higher prevalence of known risk factors for PSCC such as human papillomavirus (HPV) infection, zoophilia, and lack of circumcision in childhood.^4-7^

Multimodal treatment with neoadjuvant chemotherapy (NAC) followed by surgery is the standard approach for locally advanced PSCC. In a retrospective trial from MD Anderson Cancer Center with PSCC patients and lymph node involvement, NAC with paclitaxel, ifosfamide, and cisplatin (TIP) showed an overall response rate (ORR) of 60% and half of the patients who had response was alive in 5 years.^8^ Otherwise, the prognosis of metastatic PSCC (mPSCC) is poor, with platinum-based chemotherapy being the standard systemic treatment associated with an ORR of 30%-40% and an overall survival (OS) around 12 months.^9-12^

In recent years, with advances in molecular and genomic assessments of tumors, efforts have been made to better understand the tumor microenvironment and genomic profile (GP) of PSCC in search of more effective treatment that can improve oncological outcomes. Ali et al evaluated with next generation sequencing (NGS) 20 PSCC tumor samples and found clinically relevant genomic alterations such as EGFR amplification and BRCA2 insertions/deletions.^13^ Thus, targeted therapy with EGFR inhibitors were evaluated in first- and second-line treatment setting showing an ORR of 32.1% and 27.3%, respectively.^14,15^ Considering that the immune checkpoint inhibitor (ICI) pembrolizumab is approved for the treatment of patients with metastatic solid tumors with high tumor mutational burden (TMB), microsatellite instability-high (MSI-H) or mismatch repair-deficient (dMMR), predictive biomarkers for immunotherapy are being investigated in PSCC.^16,17^ In this context, there are data showing that around 14% and 60% of the patients with PSCC may present high TMB and PD-L1 expression, respectively.^18,19^

In this study, we sought to investigate the PSCC genomic profile of patients from a region where this neoplasm is more prevalent and identify prognostic and predictive biomarkers that could be used in further clinical trials.

## Methods

This study was conducted following the Strengthening the Reporting of Observational Studies in Epidemiology (STROBE) statement.^20^

### Study population

Patients with PSCC were retrospectively identified from 3 Brazilian hospitals (Hospital São Lucas, Universidade Federal do Maranhão [UFMA] e Oncocentro). We included patients aged ≥18 years with a histologically confirmed diagnosis of PSCC, locally advanced, or metastatic disease, treated with at least one prior systemic chemotherapy, being dead at the time of the inclusion in this study and available formalin-fixed paraffin-embedded blocks. Patients with non-squamous or mixed histologies, without date of death or lost the follow-up were excluded. The sample size was defined according to the number of NGS assessments previously made available by Foundation Medicine Inc.

### Data extraction

We retrospectively collected clinical-pathological data from patient’s paper and electronic charts such as age, race, smoking, circumcision status, surgery, site of tumor tissue, tumor histological grade, status of angiolymphatic invasion, sites of metastases, type and lines of systemic chemotherapy, and date of death.

### Genomic and molecular analysis

Comprehensive GP was performed using a NGS assesment using a targeted panel (Foundation One DX) where 324 somatic genes and genomic signatures were analyzed.^21^ TMB was calculated as the number of somatic mutations per megabase (mut/Mb) in a section of DNA. Low TMB was defined as lower than 10 mut/Mb and high TMB as 10mut/Mb or higher. The evaluation of MSI-H or microsatellite stability (MSS) was based on genome-wide analysis of 95 microsatellite loci. PD-L1 expression was evaluated by immunohistochemistry (IHC) using the 22C3 pharmDX antibodies on Dako Autostainer Link 48 and interpreted using the combined positive score (CPS). CPS was calculated as follow: CPS = 100 × %PD-L1 staining cells (tumor cells, lymphocytes, and macrophages)/total % of viable tumor cells.^22^ No PD-L1 expression was defined as CPS lower than 1% and positive PD-L1 expression as CPS 1% or higher. HPV 16/18 status was determined by IHC using the Ventana XT autostainer kit to evaluate p16 overexpression.

### Study endpoints

The primary objective is to identify and describe genomic alterations known to be functional or pathogenic according to the catalogue of somatic mutation in cancer (COSMIC)^23^ and their association with progression-free survival (PFS) and OS. Secondary endpoints include determination of TMB, PD-L1, and HPV status and their association with the identified genomic alterations, PFS and OS. OS was calculated from the time from diagnosis of the metastatic disease to death of any cause. PFS was defined as the time from the start of first-line therapy to progression or death from any cause.

### Statistical analysis

Baseline characteristics and genomic alterations were summarized by descriptive statistics. Time-to-event analysis was performed using the Kaplan-Meier method and comparisons between groups were performed using the stratified log-rank test. The significance level was set at 5%. All analyses were conducted using SAS version 9.4 (SAS Institute).

### Ethical considerations

This study was conducted in accordance with the “Declaration of Helsinki” and approved by local ethics committees as well as the national ethics committees (protocol number: 5.123.045). Due to the retrospective nature of the study and all patients being dead, informed consent was waived by local IRBs.

## Results

### Study population

**Figure 1.**
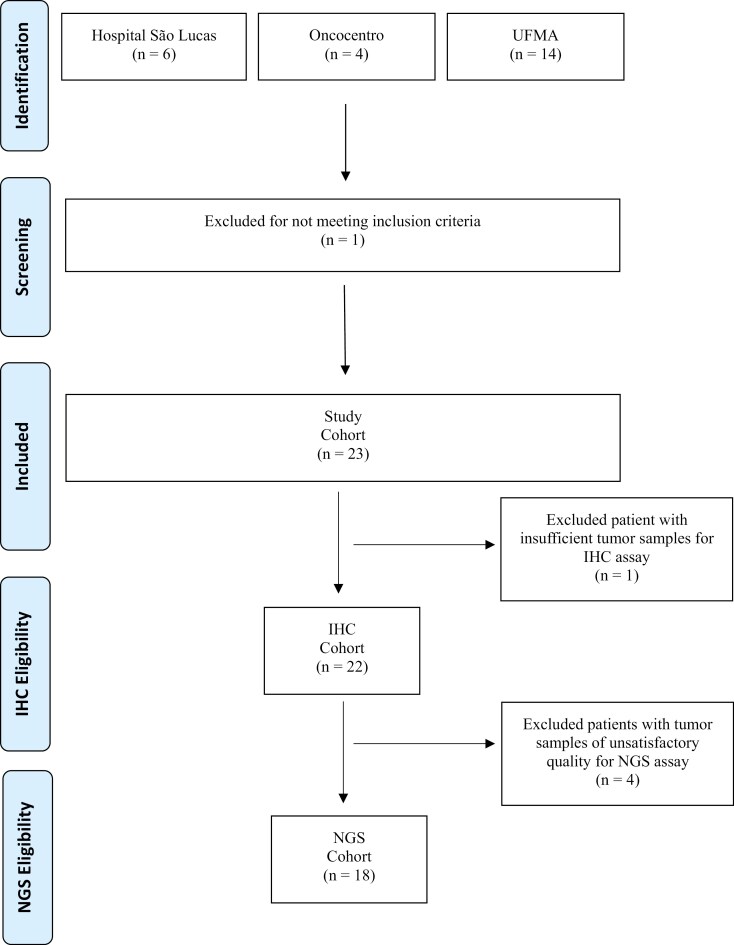
Patients identification and selection.

**Figure 2.**
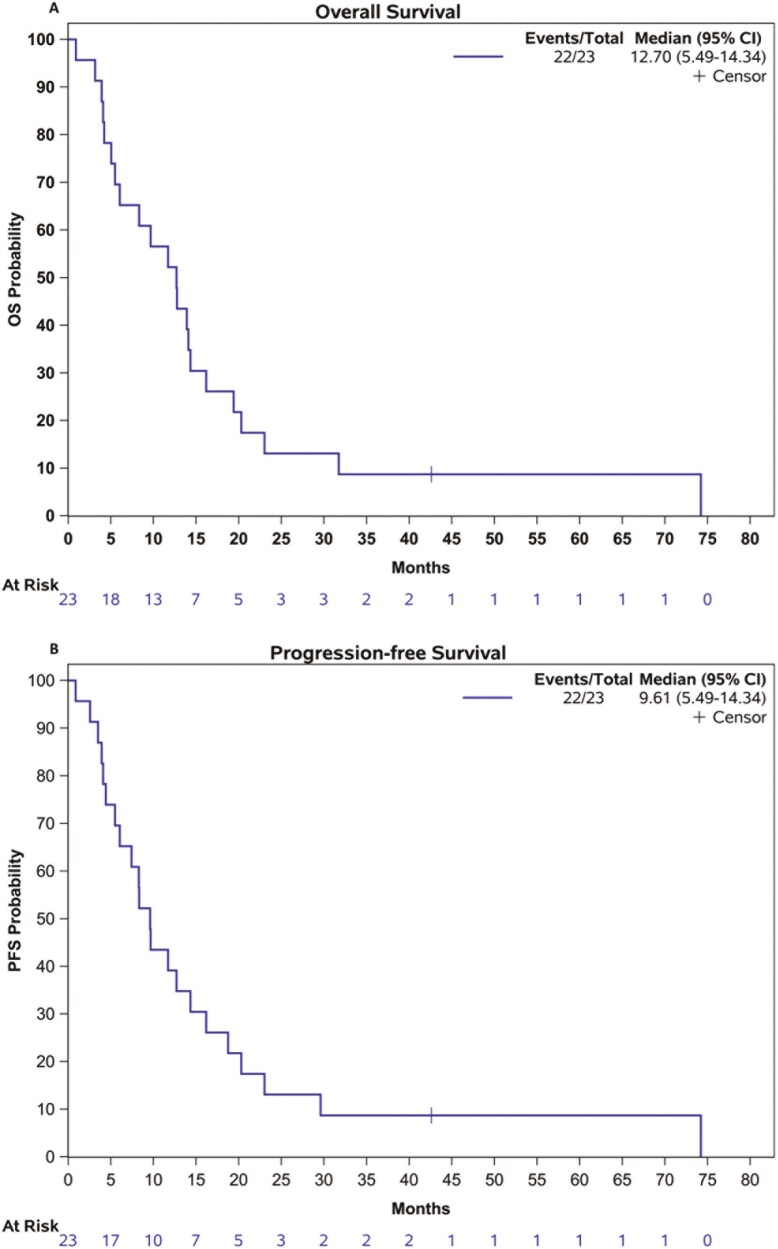
Overall survival (A) and progression-free survival (B) from the metastatic diagnosis.

We initially identified 24 patients with mPSCC, however, one patient was alive and was excluded (details of the selection and inclusion in Figure 1). For the entire cohort (*n* = 23), the median age was 53.7 (range: 18-36) years; 12 patients (52.2%) had intermediate histological tumor grade, 16 patients (69.9%) had no angiolymphatic invasion and 8 patients (36.4%) had metastatic disease at diagnosis. Regarding systemic treatment, 16 patients (69.6%) received first-line chemotherapy as follows: 9 patients (56%) received platinum-based chemotherapy, 4 patients (25%) were treated in a clinical trial with platinum-based chemotherapy plus pembrolizumab, and 3 patients (18.7%) received single agent chemotherapy. Only 1 patient (4.3%) received second-line treatment. The 7 patients (30.4%) without systemic treatment received best supportive care (clinical characteristics are summarized in Table 1). With a median follow-up of 14.3 months (95% CI: 9.7-20.1) the median OS and PFS was 12.7 (95% CI: 5.4-14.3), and 9.6 months (95% CI: 5.4-14.3), respectively (Figure 2A and B).

**Table 1.** Baseline characteristics of entire cohort.

Characteristics	Cohort (*N* = 23)	HPV positive (*N* = 9)	HPV negative (*N* = 13)
Age (range)	53.7yrs (18-86)	56.8yrs (30-86)	52.3yrs (18-71)
TNM classification			
IIB	3 (13.6)	2 (22.2)	1 (7.7)
IIIA	1 (4.5)	0 (0.0)	1 (7.7)
IIIB	10 (45.5)	3 (33.3)	6 (46.2)
IV	8 (36.4)	4 (44.4)	4 (30.8)
Missing	1 (4.5)	0 (0.0)	1 (7.7)
Circumcision			
No	14 (60.9)	7 (77.8)	6 (46.2)
Unknown	9 (39.1)	2 (22.2)	7 (53.8)
Site of tumor tissue			
Primary	20 (87)	9 (100)	10 (76.9)
Metastatic	3 (13)	0 (0.0)	3 (23.1)
Tumor histologic grade			
Well	3 (13)	2 (22.2)	1 (7.7)
Intermediate	12 (52.2)	4 (44.4)	7 (53.8)
Poor	8 (34.8)	3 (33.3)	5 (38.5)
Vascular lymphatic invasion			
Positive	4 (17.4)	3 (33.3)	0 (0.0)
Negative	16 (69.6)	6 (66.7)	10 (76.9)
Not reported	3 (13)	0 (0.0)	3 (23.1)
Site of metastatic disease			
Regional lymph nodes	19 (82.6)	6 (66.7)	12 (92.3)
Non-regional lymph nodes	1 (4.3)	0 (0.0)	1 (7.7)
Visceral	2 (8.7)	2 (22.2)	0 (0.0)
Unknown	1 (4.3)	1 (11.1)	0 (0.0)
First-line systemic treatment	16 (69.6)	5 (55.6)	10 (76.9)
CDDP + 5FU + pembrolizumab	4		
CDDP + 5FU + docetaxel	3		
CDDP + ifosfamide + paclitaxel	2		
CDDP + 5FU	2		
CDDP + capecitabine	1		
Carboplatina + paclitaxel	1		
Paclitaxel or docetaxel	3		
Second-line systemic treatment	1 (4.3)	0 (0.0)	1 (7.7)
MTX + vincristine + bleomycin	1		

Abbreviations: CDDP, cisplatin; 5FU, 5-fluouracil; MTX, methotrexate.

### NGS evaluation

Excluding patients with unsatisfactory quality of the tumor samples (low DNA content or poor tumor purity), 18 patients were eligible for NGS evaluation; 17 patients (94.4%) had the tumor samples from the primary tumor, 9 patients (50%) had intermediate histological tumor grade, and 12 patients (66.7%) received first-line systemic treatment for metastatic disease (clinical characteristics are summarized in Supplementary Table S1).

**Figure 3.**
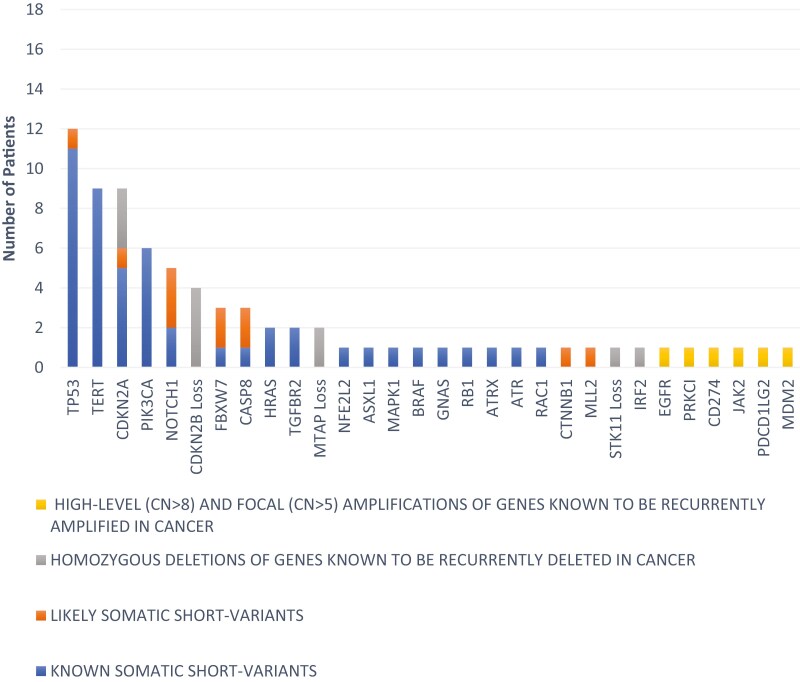
Genomic alterations identified by NGS.

Regarding the genomic alterations, TP53, CDKN2A, TERT, NOTCH 1, and CDKN2B loss were identified in 12 (66.7%), 9 (50%), 9 (50%), 5 (27.8%), and 4 (22.2%) patients, respectively. Potentially targetable genomic alterations such as PIK3CA mutation, EGFR amplification, and BRAF mutation were identified in 6 (33.3%), 2 (11.1%), and 1 (5.6%) patient, respectively. Details of the targetable alterations are summarized in Supplementary Table S2 and all genomic alterations identified are illustrated in Figure 3. MSI status was undetermined in 11 (61.1%) patients and MSS in 6 (33.3%) patients. There was no high TMB patients identified. The median TMB level was 3.85 mut/Mb (range: 0-8.83).

**Figure 4.**
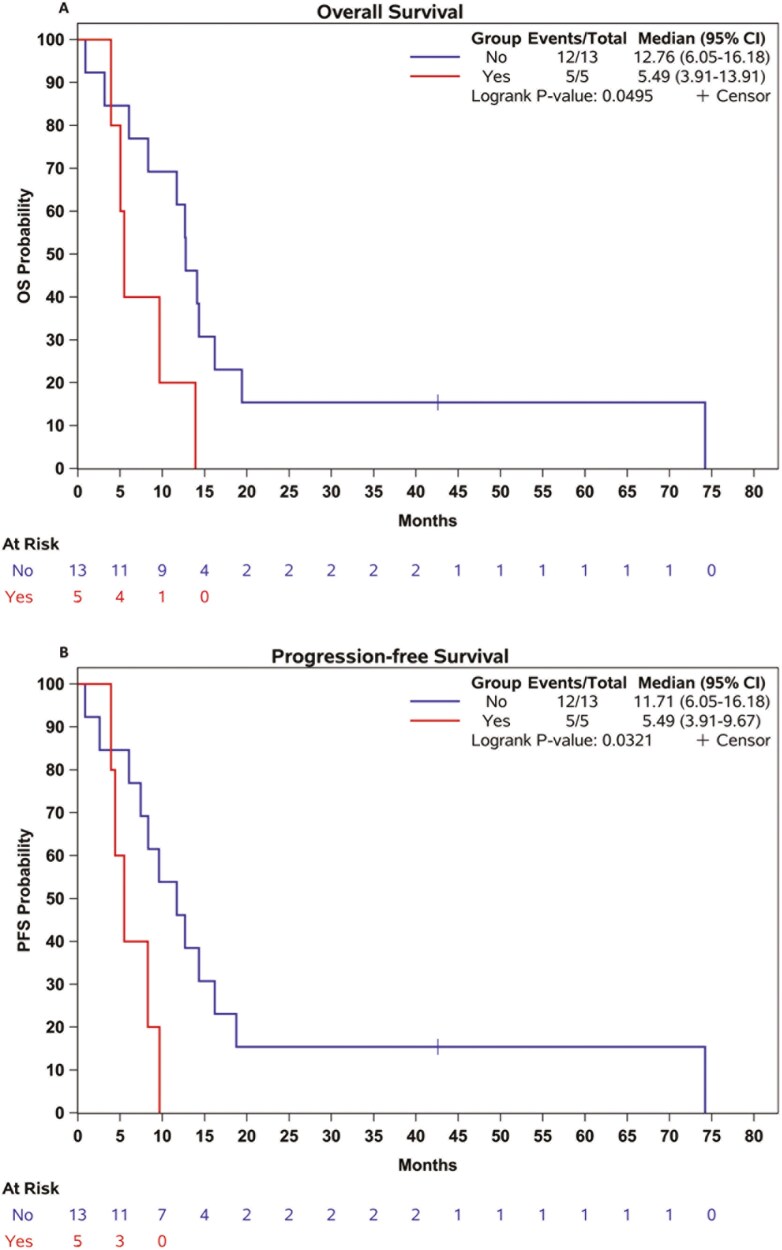
Overall survival (A) and progression-free survival (B) from metastatic diagnosis by NOTCH1.

In the context of prognosis, NOTCH1 alteration was associated with significant poor survival (mOS = yes: 5.4, 95%CI 3.9-13.9, vs no: 12.7 months, 95%CI 6.0-16.1, *P *= 0.04; mPFS = yes: 5.4, 95%CI 3.9-9.6, vs no: 11.7 months, 95%CI 6.0-16.1, *P* = 0.03; Figure 4A and B). OS and PFS according to the genomic alterations are summarized in Table 2 and Supplementary Figures S1-S5.

**Table 2.** Overall and progression-free survival according to genomic alterations (significant *p* values are highlighted in bold) .

Genomic alteration	Status	mOS (95%CI)		mPFS (95%CI)	
TP53	Yes	12.7 mo (3.9-16.1)	*P < 0.82*	8.3 mo (2.5-74.2)	*p* = 0.86
	No	10.0 mo (3.9-16.1)			
CDKN2A	Yes	12.7 mo (3.9-16.1)	*P* = .11	12.7 mo (0.85-74.2)	*P* = .10
	No	9.6 mo (3.1-14.1)		8.3mo (2.5-11.7)	
TERT	Yes	12.7 mo (0.8-16.1)	*P* = .98	7.4 mo (0.85-16.1)	*P* = .65
	No	11.7 mo (3.1-19.4)		9.6 mo (2.5-18.7)	
PIK3CA	Yes	12.2 mo (0.8-74.2)	*P* = .90	12.2 mo (0.8-74.2)	*P = *.58
	No	11.2 mo (3.9-16.1)		8.3 mo (3.9-16.1)	
CDKN2B Loss	Yes	14.4 mo (5.4-74.2)	*P* = .33	14.4 mo (5.4-74.2)	*P = *.23
	No	10.6 mo (3.4-14.1)		8.3 mo (3.9-11.7)	
NOTCH1	Yes	5.4 mo (3.9-13.9)	***p = .04***	5.4 mo (3.9-9.6)	***P* = .03**
	No	12.7 mo (6.0-16.1)		11.7 mo (6.0-16.1)	

Abbreviations: mOS = median overall survival; mPFS = median progression-free survival; CI = confidence interval; mo = months.

### IHC evaluation

Excluding 1 patient with insufficient tumor sample, IHC was performed in 22 patients. PD-L1 expression (CPS ≥ 1%) was identified in 14 patients (63.6%) and HPV status was negative in 13 patients (59%). Regarding the prognosis, there was non-statistically significant survival difference associated with PD-L1 expression (mOS = yes: 11.2, 95%CI 3.9-14.3, vs no: 14.4 months, 95%CI 4.0-23.0, *P *= .44; mPFS = yes: 7.8, 95%CI 3.4-14.3, vs no: 14.4 months, 95%CI 4.0-23.0, *P* = .29; Supplementary Figure S6) and HPV status (mOS = positive: 11.7, 95%CI 0.8-74.2, vs negative: 12.7 months, 95%CI 5.0-16.1, *P *= .54; mPFS = positive: 11.7, 95%CI 0.8-7.2 vs negative: 9.6 months, 95%CI 4.4-16.1, *P* = .50; Supplementary Figure S7).

### Genomic findings according to HPV status

**Figure 5.**
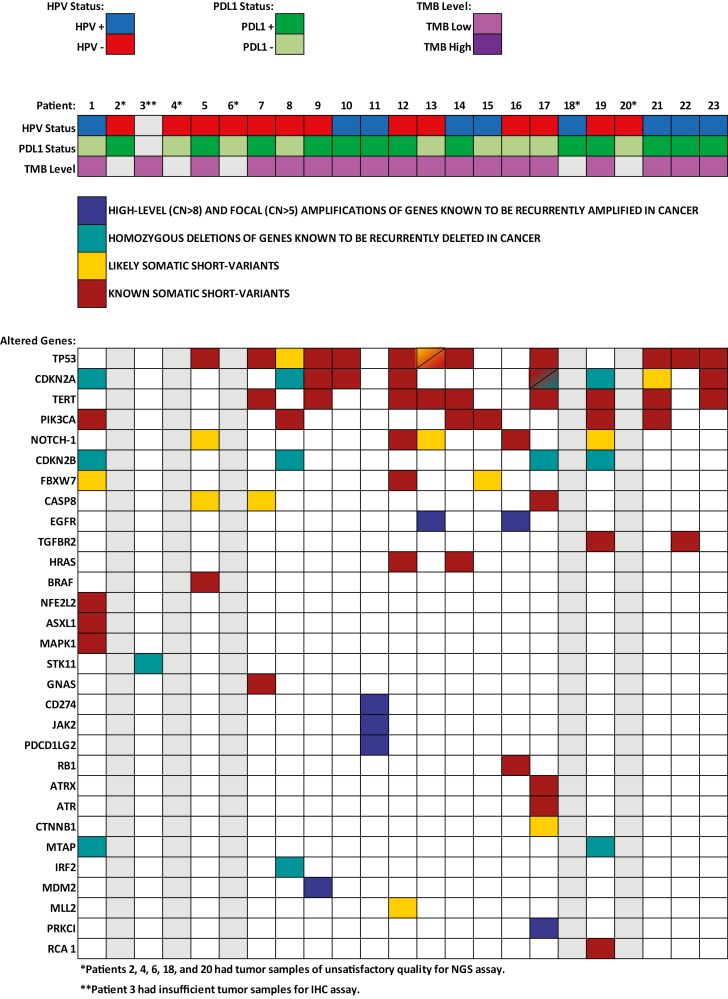
Genomic profiling according to HPV status.

Of the 18 tumor samples submitted to NGS evaluation, it was not possible to perform IHC analysis (PD-L1 and p16 expression) in one sample. Thus, considering the HPV status in 17 tumor samples, positive PD-L1 expression (CPS ≥ 1%) was identified in 87.5% and 77.8% of HPV-positive and HPV negative patients, respectively. Regarding molecular evaluation by NGS more HPV negative patients had TP53, CDKN2A TERT, and CDKN2B loss alterations. In this context, NOTCH1 alteration was only identified in HPV negative patients. The potentially targetable molecular alterations EGFR and BRAF were only identified in HPV-negative patients, otherwise more HPV-positive patients had PIK3CA alteration. The genomic profiling according to HPV status is summarized in Figure 5 and Supplementary Table S3.

## Discussion

To the best of our knowledge, this is the first study to characterize the genomic profile of PSCC in patients exclusively from a developing country, where the incidence rate of this neoplasm is up to six times higher than in developed countries and there are uncertainties related to the divergence in genomic signatures in different geographical and cultural patient populations.

A meta-analysis including 4199 patients with PSCC demonstrated that half of the cases of PSCC are associated with HPV infection.^5^ Giuliano et al., demonstrated in an observational study with genital tissue of 1160 men from US, Mexico, and Brazil, that around 70% of Brazilian men have HPV infection in genital tissue.^6^ Despite this evidence, we identified in this cohort that 59% of patients had HPV-independent PSCC. A plausible reason for this is the indirect IHC evaluation methodology that we used to identify HPV infection. The p16 overexpression has 67% and 91% sensitivity and specificity, respectively, for defining HPV infection status in PSCC.^24^ A retrospective study using the direct HPV molecular detection methodology with polymerase chain reaction (PCR) in 113 samples of PSCC identified high-risk HPV16 infection in 75.5% of the cases. However, using IHC evaluation in the same sample the p16 overexpression was identified in only 26% of the PSCC samples.^25^ Another reasonable hypothesis is that other risk factors such as lack of circumcision, poor penile hygiene, and chronic dermatoses would be more preponderant for the development of PSCC in our population. It is important considering that HPV-induced and independent PSCC have different tumor microenvironment (TME), PD-L1 expression, TMB as well as genomic profiling.^26,27^

PD-L1 expression is a predictive biomarker of response to ICI in some neoplasms such as melanoma, lung, and gastric cancer.^28^ In PSCC, retrospective trials from North America using different antibodies to evaluate PD-L1 expression and CPS cutoff of ≥5% and ≥1%, showed positive PD-L1 expression in 62.2% and 40% of the patients, respectively, not related to HPV infection, and associated with a decreased cancer-specific survival.^19,29^ Otherwise, a cohort of 40 patients with localized PSCC from Brazil using the same antibody and CPS cutoff of our study, showed PD-L1 expression in 51.4% of the patients, an association with p16 expression and no difference in survival.^30^ In this context, the PD-L1 expression (CPS ≥ 1%) observed in our study is quite similar to the data from North America cohorts and had no impact in PFS and OS. As observed in the previously cited Brazilian study, in our cohort positive PD-L1 expression was more likely in HPV-positive patients. This is in line with a study that evaluated 9887 tumor samples of different types of cancer and demonstrated that viral-associated tumors are more likely to have PD-L1 expression.^31^

TMB is a predictive biomarker of efficacy to treatment with ICI in solid tumors.^16^ The prevalence of TMB is different across tumors, with small cell lung cancer, dMMR colorectal, and gastric cancer presenting the highest median of TMB level.^32^ Therefore, the assessment of TMB in PSCC has been investigated. A Chinese study with 20 PSCC tumor samples showed that 85% of the cases had TMB < 10 mut/Mb with and the median TMB level was 7.4 mut/Mb.^33^ Recently, Necchi et al. in an observational study with 397 patients with PSCC from the Foundation Medicine Inc (FMI) database also demonstrated that 85.5% of the cases had TMB < 10 mut/Mb, however in this large cohort they identified 10.1% and 4.5% of the patients presenting high (10-19 mut/Mb) and very-high TMB (≥ 20 mut/Mb), respectively.^18^ In this way, Nazha et al. in a retrospective study with 108 PSCC tumor samples identified high TMB in 10.7% of the cases.^34^ In our study all subjects had TMB < 10 mut/Mb and a low median TMB level of 3.85 mut/Mb. Considering the small sample size of our cohort any inference must be made with caution and investigation on TMB in PSCC in regions where the disease is more prevalent must continue to be carried out.

Regarding the somatic genomic alterations, TP53, CDKN2A, TERT, PIK3CA, NOTCH1, and EGFR were identified in more than 10% of our cohort. These genomic alterations were also identified in the largest genomic landscape of PSCC from FMI database and TP53, CDKN2A, PIK3CA, and NOTCH1 were listed as top gene alterations of PSCC in COSMIC database.^18,23^ CDKN2B loss was identified in 22.2% of our cohort and this genomic alteration was not observed in the FMI and COSMIC database as well as other genomic trials.^13,34-36^ CDKN2B is a tumor suppressor gene that inhibits the activation of CDK kinases. CDKN2B loss may be present in up to 8% of different types of cancer including glioblastoma, pancreatic, and lung adenocarcinoma.^37,38^ In urinary tract tumors, CDKN2B loss varies by histology and tumor site, being more frequent in urothelial cancer (up to 30%) and in SCC (up to 39%).^39^ From the point of view of new therapeutic options for PSCC, it is important to mention that CDKN2B loss in patients with non-small cell lung cancer is associated with worse efficacy and survival with ICI alone treatment, but not with ICI plus chemotherapy treatment.^40^

In this study, NOTCH1 alteration was exclusively identified in HPV-snegative patients and was the only genomic alterations associated with a significant decreased survival. NOTCH1 is part of 4 transmembrane receptors protein that are involved in cell differentiation, proliferation, and apoptosis.^41^ NOTCH1 mutations have been identified in squamous cell carcinomas (SCC) of different organs.^42^ Like PSCC, vulvar SCC has HPV infection as a risk factor and is also divided into HPV associated or independent tumor.^43,44^ However, in vulvar SCC NOTCH1 mutations is correlated to HPV infection and is not associated with shorter survival.^45^ In the context of PSCC, Chahoud et al. in a trial with 34 PSCC tumor samples observed NOTCH1 alterations in 70.6% of the cases, a pattern similar to that identified in head and neck squamous cell carcinoma and not correlated to HPV infection.^36,46^ In a clinical perspective, NOTCH-targeting therapeutic with different drugs has been investigated in clinical trials for various types of tumors.^47^ In this way, an observational trial with advanced prostate cancer samples presenting NOTCH1 mutation showed that cancer cell were sensitively targeted by BMS-754807, linsitinib, saracatinib, and erlotinib.^48^ In a phase 2 trial a γ-secretase inhibitor showed a response rate of 29% in patients with desmoid tumors.^49^ Rovalpituzumab teserine plus nivolumab was evaluated in a phase 2 trial with extensively treated patients with advanced small cell lung cancer, demonstrating a response rate of 30%.^50^

Considering other molecular alterations such as EGFR, which was also identified in our cohort, a retrospective study demonstrated that the combination of cetuximab with and platinum chemotherapy resulted in an ORR of 31% in 13 patients with mPC. However, cetuximab alone as a second-line treatment or beyond only 1 patient showed a partial response out of the 8 patients.^51^ Despite the seemingly limited effectiveness of cetuximab alone and in its use in more advanced lines of treatment, cetuximab and other EGFR inhibitors with platinum-based chemotherapy should be considered in future clinical trials.

## Limitations

Our study has some limitations. The retrospective design and small sample size are worthy of concern. Missing clinical data such as smoking status, history of circumcision, and zoophilia^7,52^ can lead to a less consistent interpretation of risk factors, as well as the lack of clinical data such as comorbidities and use of concomitant medications makes it difficult to adequately control confounding factors for clinical outcomes. Even considering that IHC evaluation of HPV status is widely used in clinical practice, this assessment using indirect methodology may have different sensitivity and specificity depending on the type of antibody clones for IHC of p16 as well as the degree of the cutoff staining to consider p16 overexpression.^53,54^ The fact that the tumor sample available for NGS evaluation was mostly from the primary tumor and not from the metastatic site of the PSCC should also be considered a weakness of the study, considering the genomic differences that exist between neoplasms in early and late stages, mainly in cases with a history of previous systemic treatments.^55^

## Conclusion

In summary, our genomic profiling of metastatic PSCC using a validated NGS platform and IHC evaluation is the first study to include patients exclusively from a country where this neoplasm is endemic. The somatic genomic alterations identified in our cohort are consistent with previous NGS evaluations in PSCC from developed countries. Based in treatment options for other cancers, we identified some potential targetable genomic alterations such as PIK3CA and EGFR to help the selection of patients with PSCC for future clinical trials with targeted therapies including Alpelisib and Cetuximab. The absence of high TMB and the identification of CDKN2B loss in some patients raises some concerns regarding the effectiveness and benefit of ICI treatment in this population.

## Supplementary material

Supplementary material is available at *The Oncologist* online.

oyae220_suppl_Supplementary_Tables

oyae220_suppl_Supplementary_Figures

## Data Availability

The data that support the results of this study are available on request from the corresponding author.
