# Supplementary material for: Molecular characterization of metastatic penile squamous cell carcinoma in developing countries and its impact on clinical outcomes: LACOG 2018 translational study
Source: Oncologist. 2024 Sep 2;30(2):oyae220. doi: 10.1093/oncolo/oyae220 (PMC12090351; doi:10.1093/oncolo/oyae220)
Supplement: oyae220_suppl_Supplementary_Tables [file oyae220_suppl_supplementary_tables.docx]

Supplementary Appendix

Supplemental Tables

Table S1 – Baseline Characteristics of NGS cohort

| Characteristics | NGS Cohort  (N = 18) |
| --- | --- |
| **Age (range)** | 54.3yrs (30–86) |
| **TNM Classification** |  |
| IIB | 3 (17.6) |
| IIIA | 1 (5.9) |
| IIIB | 6 (35.3) |
| IV | 7 (41.2) |
| Missing | 1 (5.6) |
| **Circumcision** |  |
| No | 12 (66.7) |
| Unknow | 6 (33.3) |
| **Site of Tumor Tissue** |  |
| Primary | 17 (94.4) |
| Metastatic | 1 (5.5) |
| **Tumor Histologic Grade** |  |
| Well | 1 (16.7) |
| Intermediate | 9 (50) |
| Poor | 6 (33.3) |
| **Vascular Lymphatic Invasion** |  |
| Positive | 4 (22.2) |
| Negative | 13 (72.2) |
| Not reported | 1 (5.6) |
| **Site of Metastatic Disease** |  |
| Regional lymph nodes | 16 (88.9) |
| Visceral | 1 (5.6) |
| Unknow | 1 (5.6) |
| **First-Line Systemic Treatment** | 12 (66.7) |
| **Second-Line Systemic Treatment** | None |

Table S2 – Details of targetable alterations

| **PIK3CA Mutation (N = 6)** | | | |
| --- | --- | --- | --- |
| **Patient** | **Nucleotide** | **AA mutation** | **Domain** |
| 1 | c.1633G>A | p.E545K | Helical |
| 2 | c.1624G>A | p.E542K | Helical |
| 3 | c.1633G>A | p.E545K | Helical |
| 4 | c.1031T>G | p.V344G | C2 |
| 5 | c.1624G>A | p.E542K | Helical |
| 6 | c.1634A>G | p.E545G | Helical |
| **BRAF Mutation (N = 1)** | | | |
| **Patient** | **Nucleotide** | **AA mutation** | **Class** |
| 1 | c.1406G>C | p.G469A | 2 |
| **EGFR Amplification (N = 2)** | | | |
| **Patient** | **Copy Number** | **Exons** |  |
| 1 | 7 | 30 of 30 |  |
| 2 | 8 | 18 of 18 |  |

| Table S3. Genomic profiling according to HPV status | | |
| --- | --- | --- |
| **Genomic Alterations** | **HPV Negative**  **(N=9)** | **HPV Positive**  **(N=8)** |
| PD-L1 positive | 7 (77.8) | 7 (87.5) |
| TMB Low | 9 | 8 |
| TP53 | 7 (77.8) | 5 (62.5) |
| CDKN2A | 5 (55.6) | 4 (50.0) |
| TERT | 6 (66.7) | 3 (37.5) |
| PIK3CA | 2 (22.2) | 4 (50.0) |
| NOTCH-1 | 5 (55.6) | 0 (0.0) |
| CDKN2B loss | 3 (33.3) | 1 (12.5) |
| FBXW7 | 1 (11.1) | 2 (25.0) |
| CASP8 | 3 (33.3) | 0 (0.0) |
| EGFR | 2 (22.2) | 0 (0.0) |
| TGFBR2 | 1 (11.1) | 1 (12.5) |
| HRAS | 1 (11.1) | 1 (12.5) |
| BRAF | 1 (11.1) | 0 (0.0) |
| NFE2L2 | 0 (0.0) | 1 (12.5) |
| ASXL1 | 0 (0.0) | 1 (12.5) |
| MAPK1 | 0 (0.0) | 1 (12.5) |
| STK11 loss | 0 (0.0) | 0 (0.0) |
| GNAS | 1 (11.1) | 0 (0.0) |
| CD274 amplification | 0 (0.0) | 1 (12.5) |
| JAK2 amplification | 0 (0.0) | 1 (12.5) |
| PDCD1LG2 amplification | 0 (0.0) | 1 (12.5) |
| RB1 | 1 (11.1) | 0 (0.0) |
| ATRX | 1 (11.1) | 0 (0.0) |
| ATR | 1 (11.1) | 0 (0.0) |
| CTNNB1 | 1 (11.1) | 0 (0.0) |
