# Supplementary material for: Molecular characterization of metastatic penile squamous cell carcinoma in developing countries and its impact on clinical outcomes: LACOG 2018 translational study
Source: Oncologist. 2024 Sep 2;30(2):oyae220. doi: 10.1093/oncolo/oyae220 (PMC12090351; doi:10.1093/oncolo/oyae220)

Supplementary Appendix

Supplemental Figures

Figure S1. Overall Survival (A) and Progression-Free Survival (B) from metastatic diagnosis by TP53


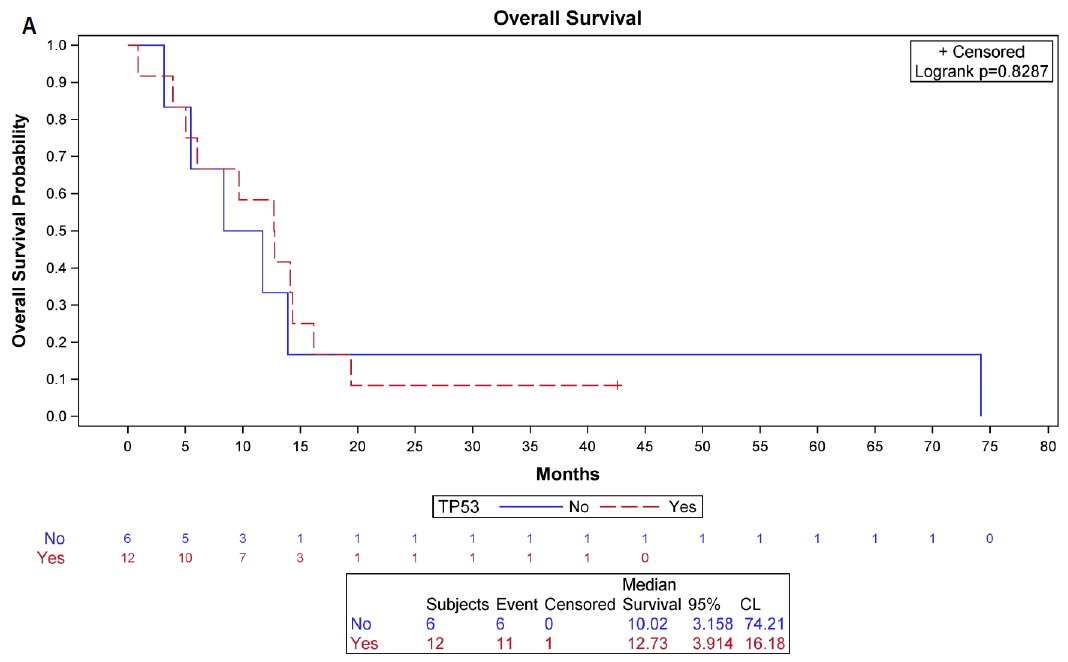


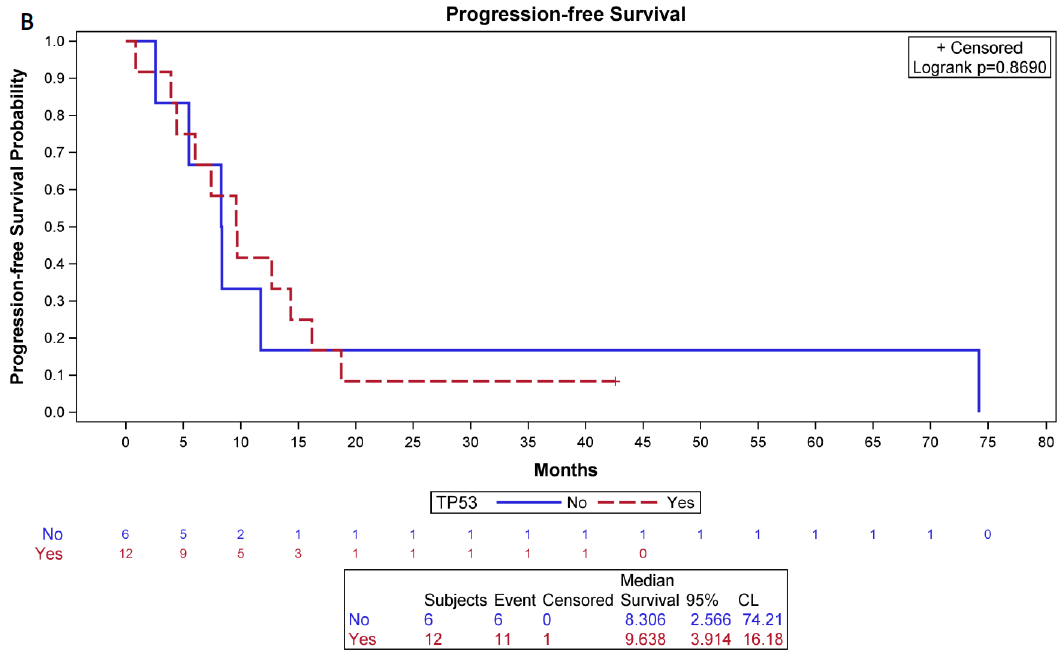


Figure S2. Overall Survival (A) and Progression-Free Survival (B) from metastatic diagnosis by CDKN2A


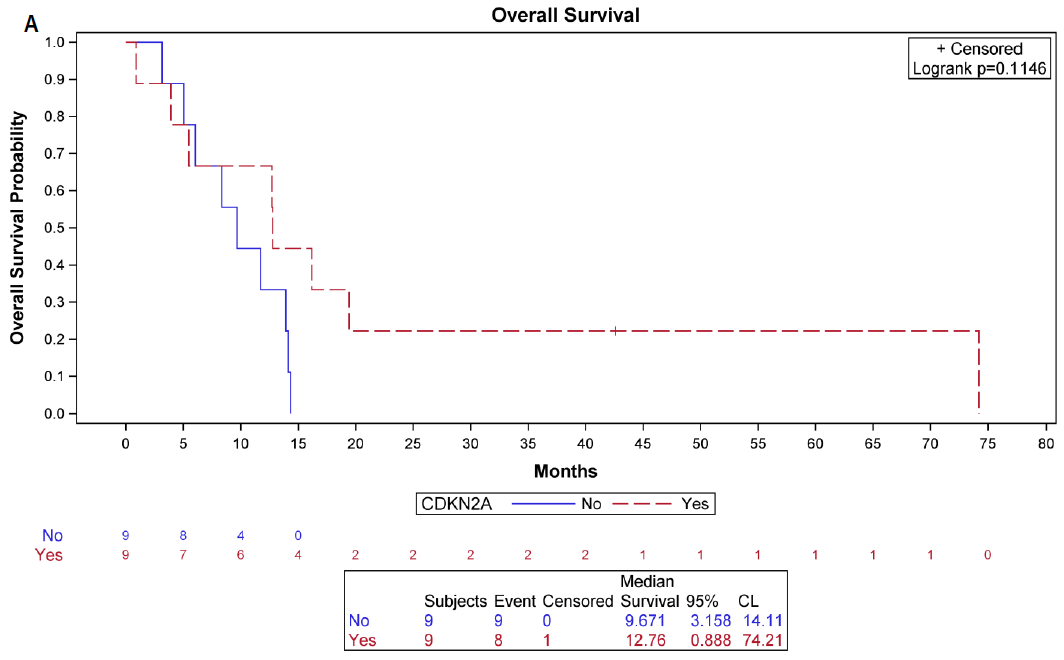


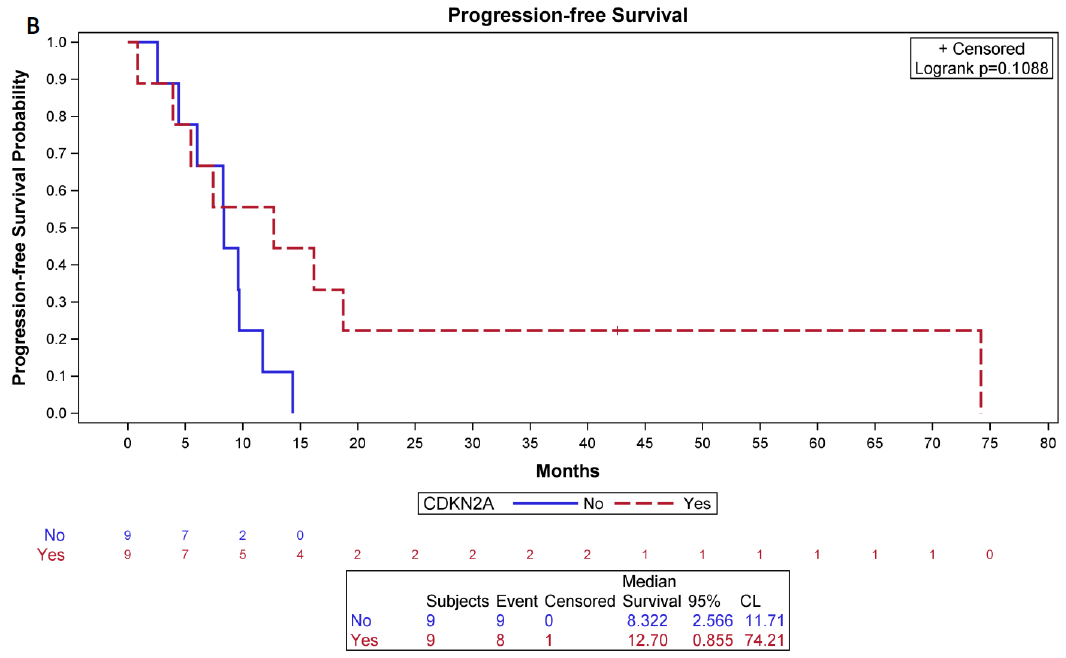


Figure S3. Overall Survival (A) and Progression-Free Survival (B) from metastatic diagnosis by TERT


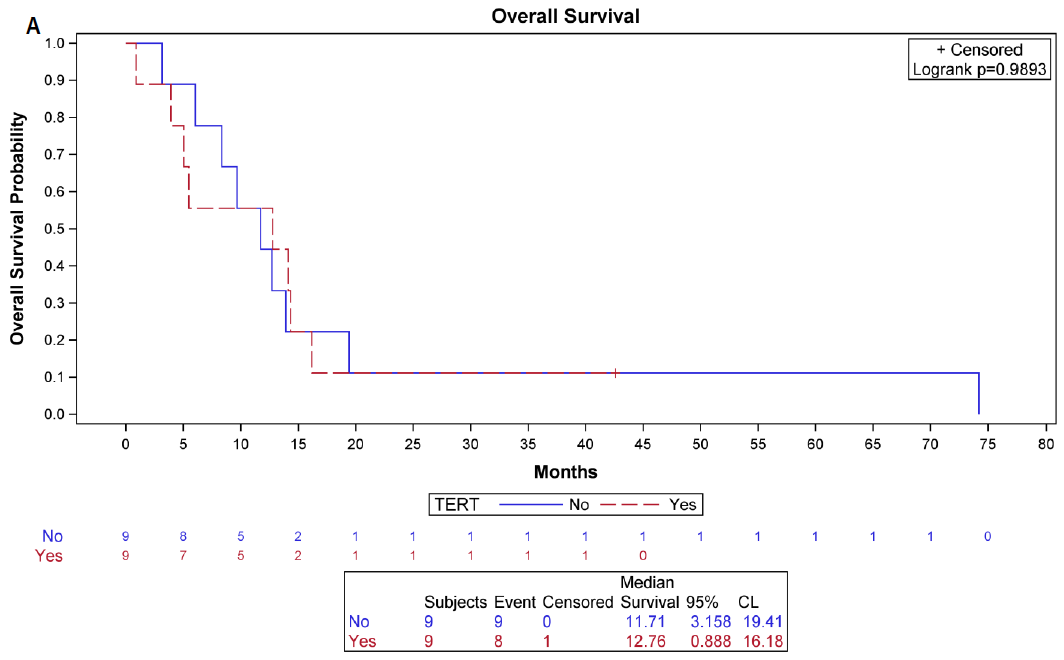


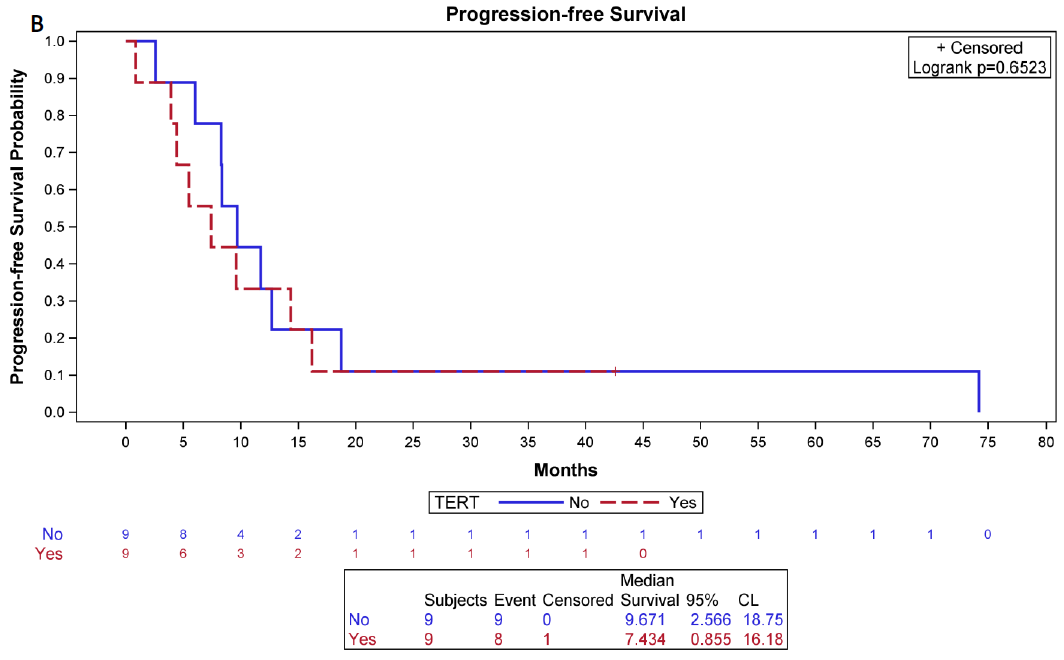


Figure S4. Overall Survival (A) and Progression-Free Survival (B) from metastatic diagnosis by PIK3CA


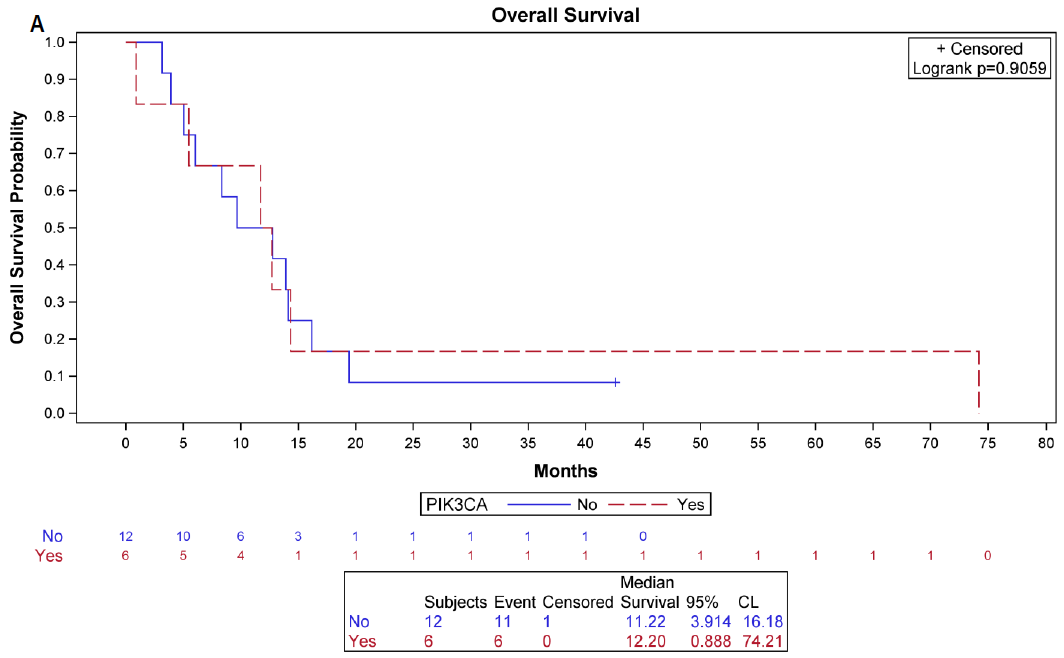


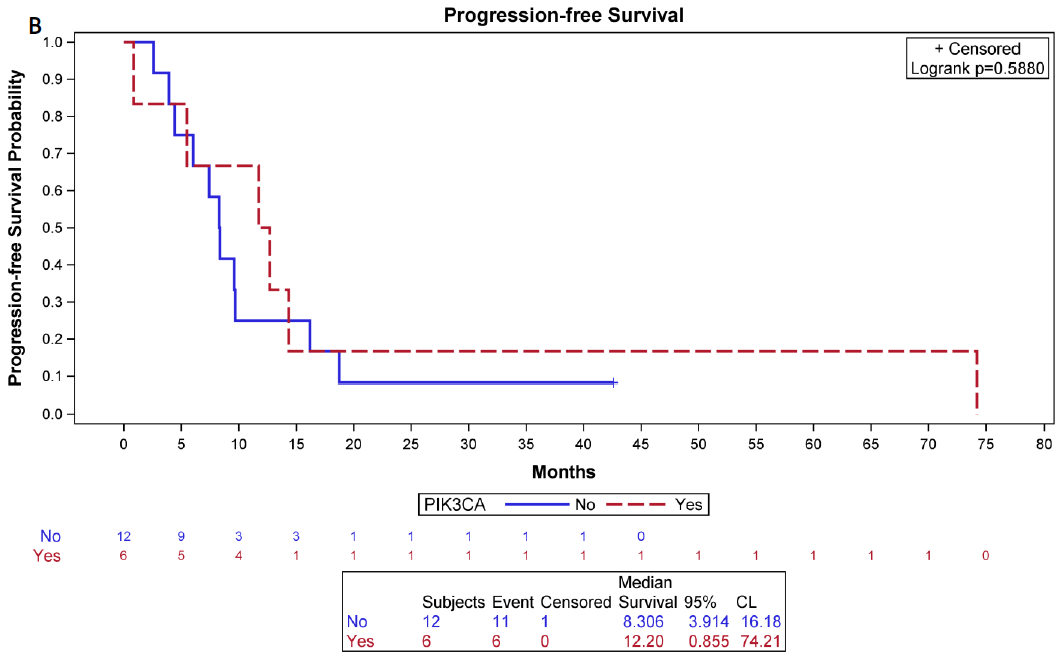


Figure S5. Overall Survival (A) and Progression-Free Survival (B) from metastatic diagnosis by CDKN2B loss


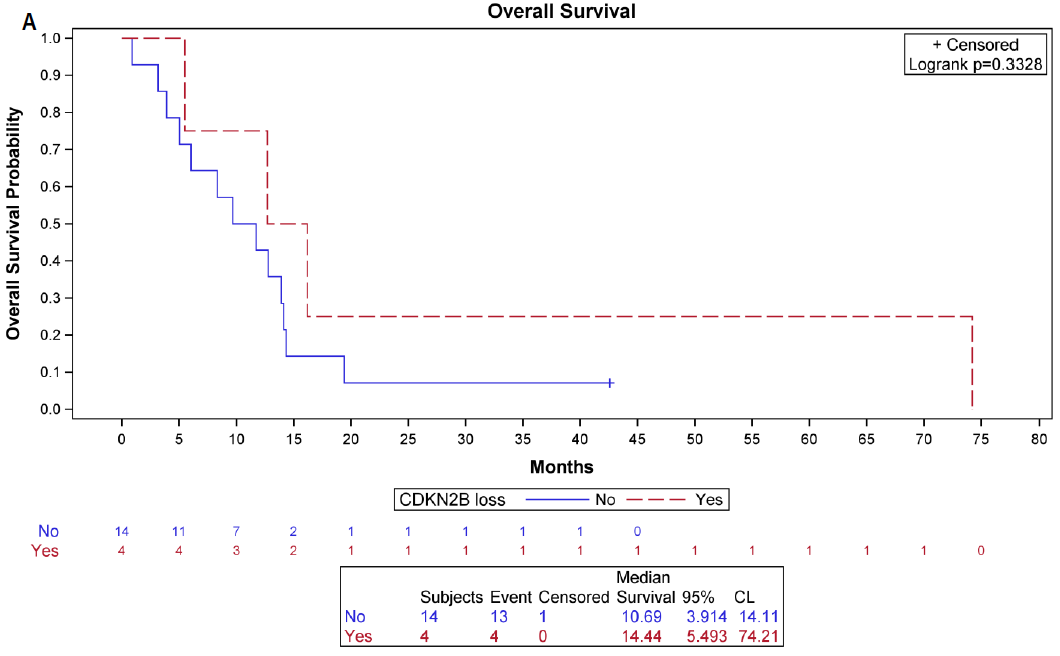


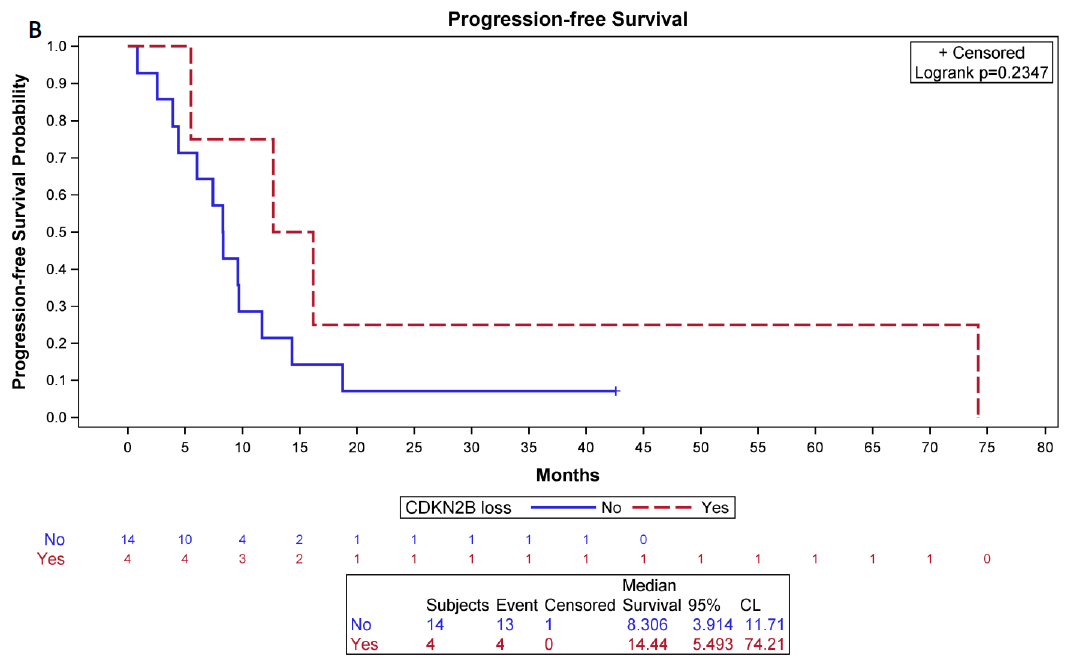


Figure S6. Overall Survival (A) and Progression-Free Survival (B) from metastatic diagnosis by PD-L1.

**A**


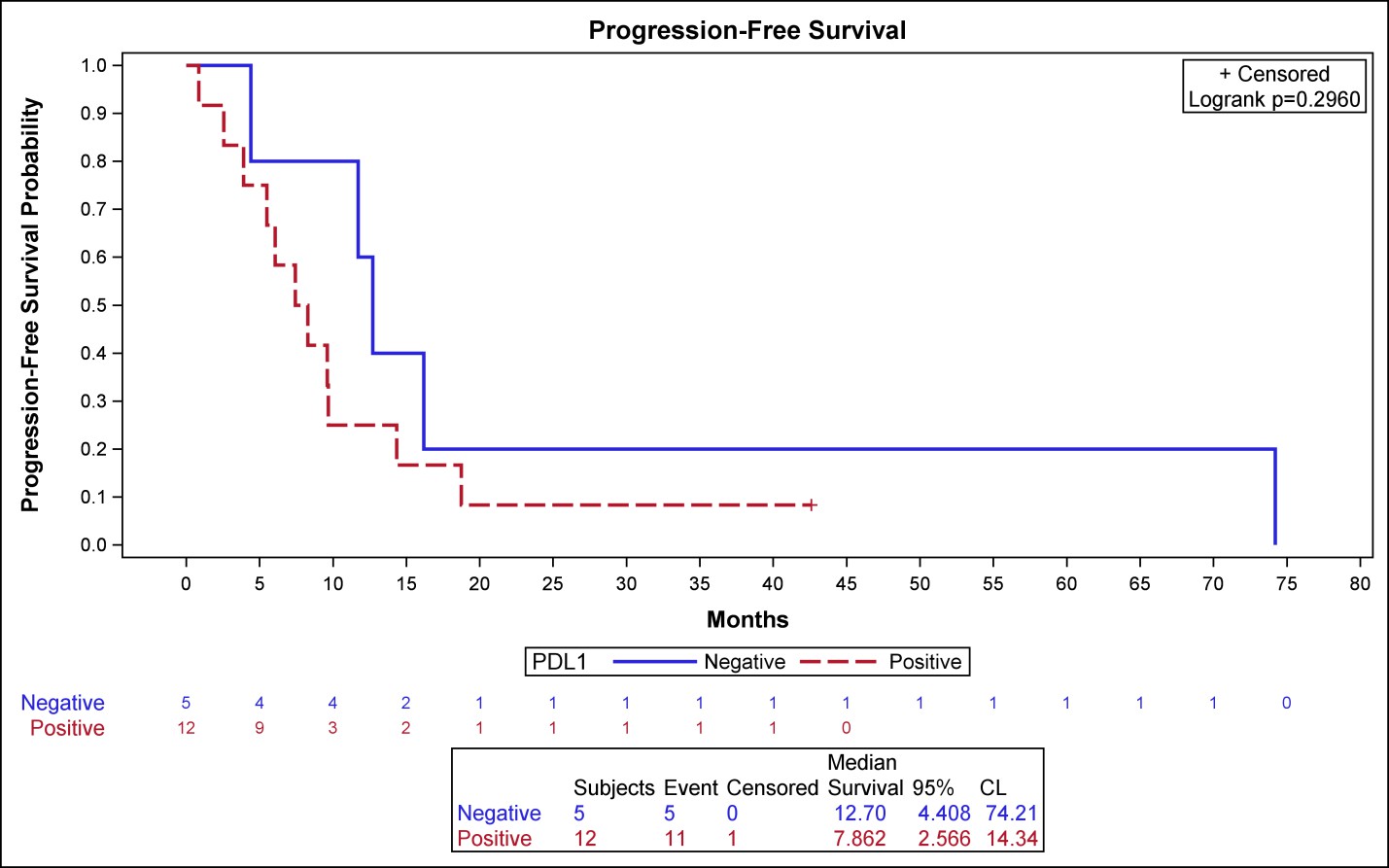

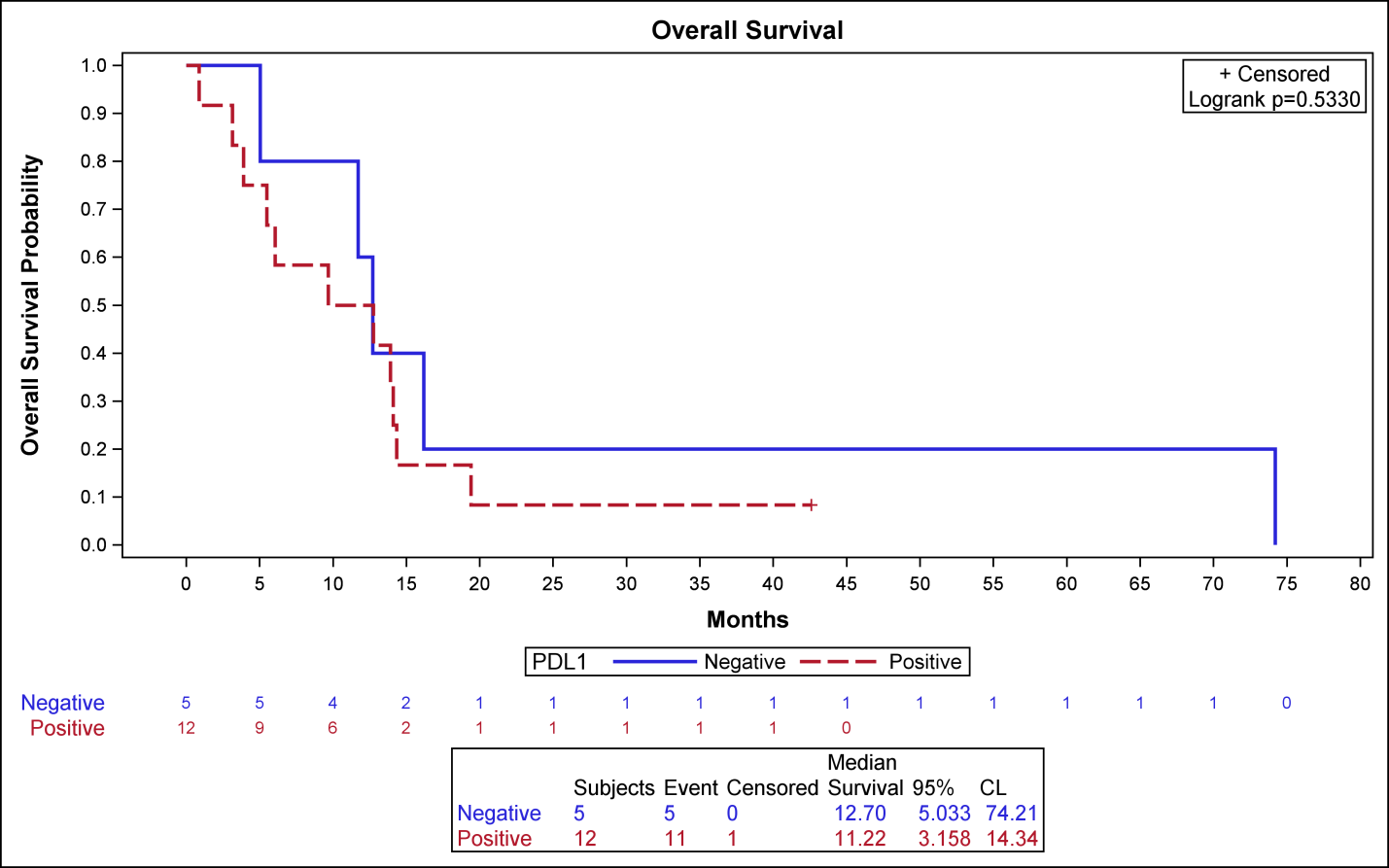
Figure S7. Overall Survival (A) and Progression-Free Survival (B) from metastatic diagnosis by HPV Status.

**B**

**A**


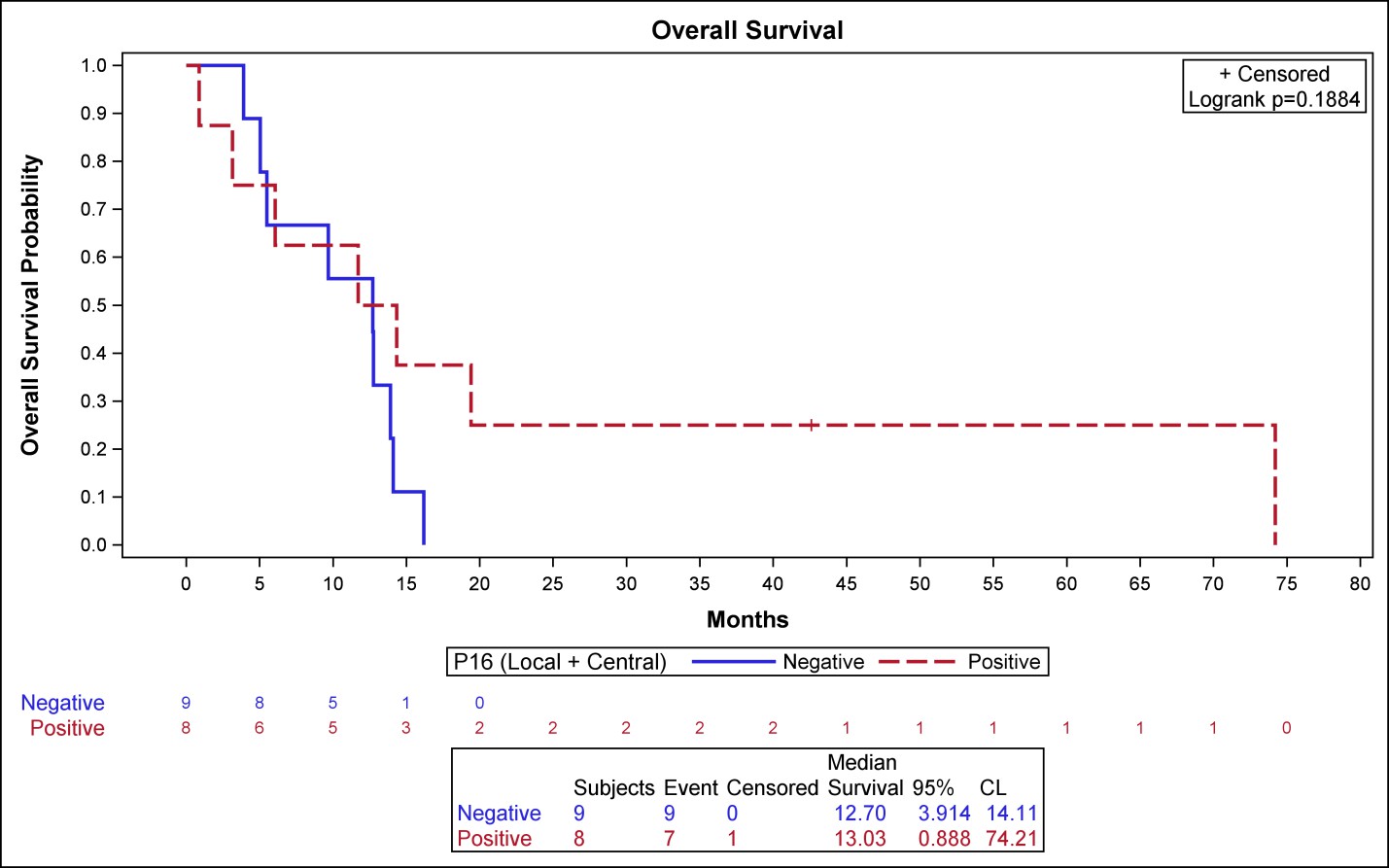


**B**


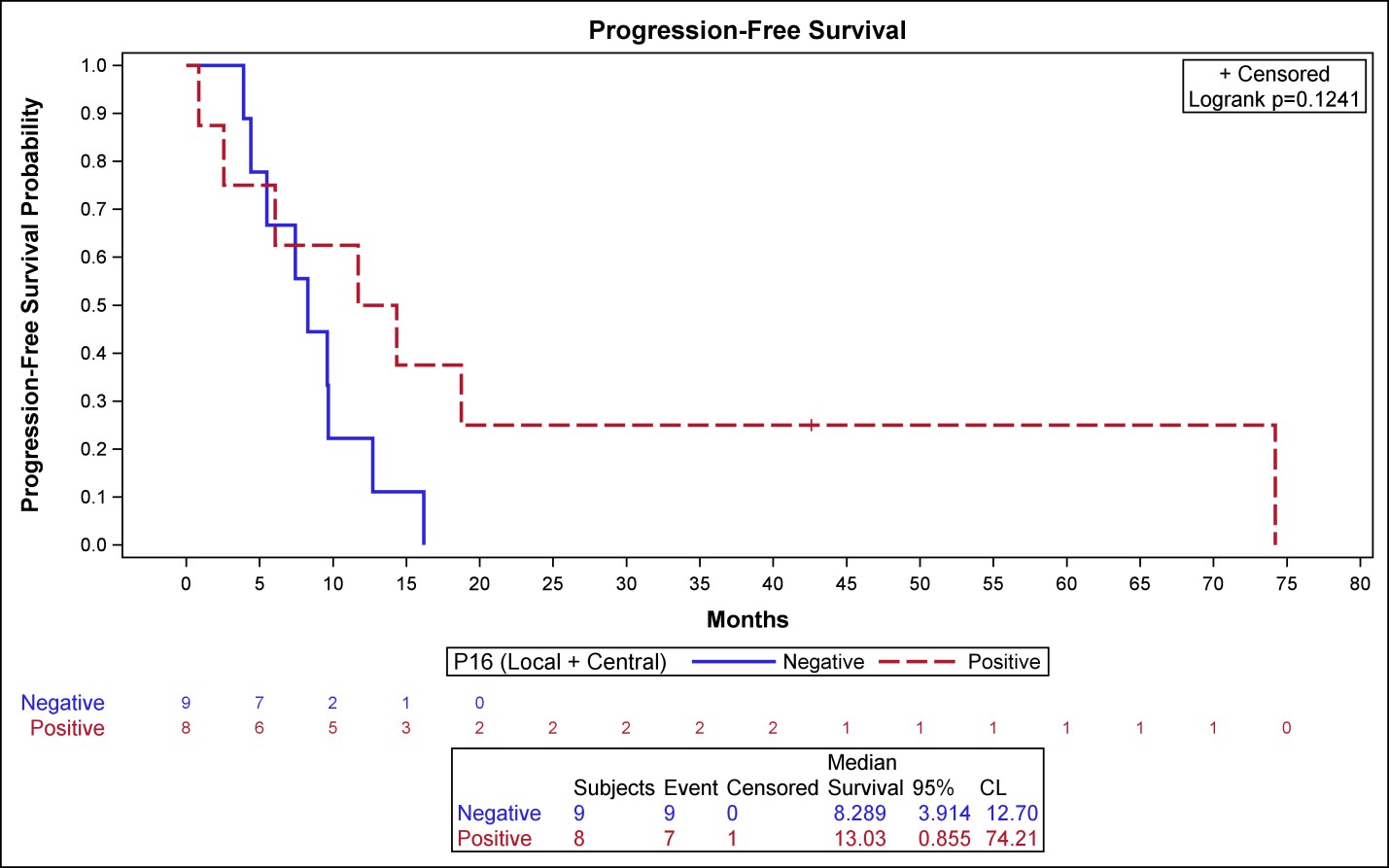

Supplement: oyae220_suppl_Supplementary_Figures [file oyae220_suppl_supplementary_figures.docx]
